# Left ventricular haematoma mimicking lateral wall myocardial infarction secondary to percutaneous coronary intervention

**DOI:** 10.5830/CVJA-2016-090

**Published:** 2017

**Authors:** Omer Senarslan, Necdet Batuhan Tamci, Umut Hasan Kantarci, Mehmet Eyuboglu, Dilsad Amanvermez Senarslan

**Affiliations:** Department of Cardiology, Medifema Hospital, Izmir, Turkey; Department of Cardiology, Izmir Atasaglik Hospital, Izmir, Turkey; Department of Radiology, Izmir Esrefpasa Hospital, Izmir, Turkey; Department of Cardiology, Special Izmir Avrupa Medicine Center, Karabaglar, Izmir, Turkey; Department of Cardiovascular Surgery, School of Medicine, Celal Bayar University, Manisa, Turkey

**Keywords:** percutaneous coronary intervention, complication, dissecting intra-myocardial haematoma

## Abstract

Dissecting intra-myocardial haematoma is a rare disease and a potentially fatal complication after cardiac surgery. Patients with previous heart surgery have more risk for dissecting intra-myocardial haematoma after percutaneous coronary intervention. Management of this issue is challenging. We describe a rare case of a 63-year-old woman with a left ventricular wall-dissecting intra-myocardial haematoma, which developed 30 minutes after percutaneous coronary intervention. The patient was treated conservatively, with a successful outcome.

## Introduction

Intra-myocardial haematoma is a rare disease and is usually associated with multiple pathologies such as myocardial infarction, chest trauma, coronary artery bypass surgery and complications of percutaneous coronary intervention (PCI), or it could occur spontaneously.^1^ Dissecting intra-myocardial haematoma (DIH) is a potentially fatal complication that is characterised anatomically and pathologically into different forms. Sub-epicardial or intra-myocardial haematoma occurs rarely and has been reported mainly in patients with previous coronary artery bypass graft (CABG) who undergo PCI.

## Case Report

**Fig. 1.**
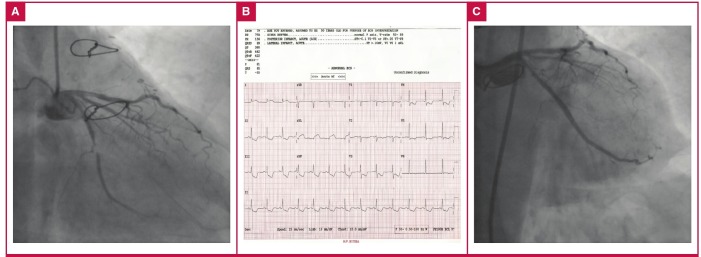
A. Coronary angiography before PCI. B. ECG after PCI. C. Control angiography. Significant stenosis is seen in the circumflex artery (A) and there is no contrast dye leakage in the control angiography (C) in the right caudal position. There is ST segment elevation in D1 and aVL deviations, suggesting a new-onset acute coronary syndrome.

A 63-year-old woman was admitted to our clinic with complaints of chest pain on effort. There was a record of CABG carried out in 2011. Coronary angiography revealed severe stenosis (99%) in the middle part of the circumflex artery (Cx) (Fig. 1A). PCI was chosen as the treatment option for the Cx lesion.

We crossed the lesion with a 0.014-inch hydrophilic guidewire PT2-LS (Boston Scientific, Natick, MA) and it was advanced distally into the Cx. We performed pre-dilatation with a coronary balloon under nominal pressure, and a 2.75 × 23-mm Xience stent (Abbott Laboratories, Abbott Park, IL, USA) was implanted under nominal pressure. The final angiogram revealed acceptable results in the Cx with no abnormal findings or contrast dye leakage.

The patient was taken to the cardiology department after PCI. Thirty minutes after the procedure, she suddenly complained of severe chest pain and discomfort. An ECG showed ST elevations in D1 and aVL deviations (Fig. 1B).

We assumed acute stent thrombosis, so we took the patient back to the catheterisation laboratory. We saw no thrombus in the stent but noticed deterioration of blood flow in the intermediate artery (Fig. 1C).

**Fig. 2.**
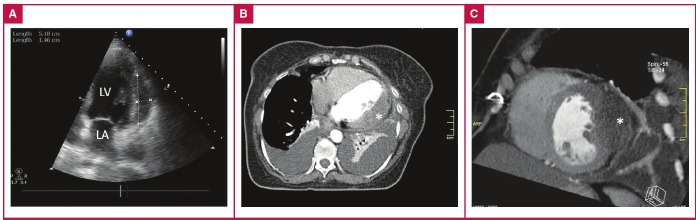
A. Transthoracic echocardiography. B, C. Axial and sagittal computed tomography sections of the heart. Intra-myocardial haematoma is seen in both echocardiography and computed tomography (asterisk). LV, left ventricle; LA, left atrium.

We checked the patient with echocardiography to see if there was a problem with the pericardium or myocardium. Two-dimensional echocardiography revealed a large 5.1 × 1.4-cm echolucent area without fluid in the pericardium (Fig. 2A). We presumed this echolucent area was a dissecting intra-myocardial haematoma in the lateral wall of the myocardium, which was compressing the intermediate artery.

Repeated bedside echocardiography revealed no change in the size of the haematoma and no deterioration in left ventricular ejection fraction or valve function on the first two days of clinical follow up. Although the left ventricular lateral wall haematoma was large enough to cause complications, it was stable, so we decided to follow the patient conservatively with standard anti-anginal therapy, including intravenous nitroglycerin and morphine sulfate. In addition, we reversed the effects of heparin with protamine sulfate.

On day three of the follow up, cardiac computed tomography revealed thickening of the lateral wall of the myocardium, a radiolucent centre without contrast dye, and bilateral pleural effusions with no pericardial effusion (Fig. 2B, C). The patient developed transient atrial fibrillation and dyspnoea on the third day. Sinus rhythm was achieved with intravenous amiodarone, and the heart failure symptoms and findings disappeared with diuretics.

The patient did not complain of chest pain or arrhythmia after the third day, and she was discharged on the sixth day of follow up. There was no haematoma in the lateral wall of the left ventricle but this part of the left ventricle was akinetic in the control echocardiography after 45 days.

## Discussion

DIH can occur as a complication of myocardial infarction, PCI and cardiac surgery.^1^ Prediction and diagnosis of DIH is very difficult after PCI and cardiac surgery. There are a few cases of DIH after PCI reported in the literature.^2^

Continued leakage of blood from the coronary artery after any kind of perforation and avulsion of the vessel can lead to dissection of the myocardium and it is characterised by dissection between the spiral planes of heart muscle, including laminated thrombi, myocytes and fibrous tissue.^2^ Self-propagation of the haematoma leads to more expansion, and it can be complicated by myocardial wall rupture.^3^

Patients with previous cardiac surgery may have a selflimiting DIH because of pericardial adhesions to the epicardium. Therefore, these patients may be protected from myocardial rupture.^4^,^5^

Since it is a rare situation, management of DIH is challenging in evidence-based medicine. Conservative management of DIH is associated with a mortality rate as high as 90 to 100%.^2^ Management strategies depend on the location and/or extent of the DIH.

Left ventricular apical location of the DIH has higher spontaneous reabsorption rates so conservative management is preferable for this position. Evacuation of the DIH and surgical repair of the myocardium are the main strategies for an unstable patient with haemodynamic impairment. However, reported surgical mortality rates range from 0–80%, presumably because of the complex patho-anatomy and friable myocardium.^2^ We decided to manage the patient conservatively due to the short period of haemodynamic instability, the size stabilised after protamine treatment, and there were no significant related complications.

## Conclusion

Since there are high mortality rates and difficulties in the management of DIH, the main treatment strategies should be based on prevention of this disease in adults. Control of the guide-wire (especially hydrophilic ones) is very important during PCI. Management of DIH should be individualised, integrating the patient’s haemodynamic stability, the size, location and extent of the DIH, and development of DIH-related complications.
